# Deletion mutation in *BSCL2* gene underlies congenital generalized lipodystrophy in a Pakistani family

**DOI:** 10.1186/1746-1596-8-78

**Published:** 2013-05-09

**Authors:** Obaid Ur Rahman, Nadeem Khawar, Muhammad Aman Khan, Jawad Ahmed, Kamran Khattak, Jumana Yousuf Al-Aama, Muhammad Naeem, Musharraf Jelani

**Affiliations:** 1 Medical Genetics and Molecular Biology Unit, Biochemistry Department, Institute of Basic Medical Sciences, Khyber Medical University, Peshawar 25000, Pakistan; 2 Pediatrics Department, Khyber Teaching Hospital, Peshawar 25000, Pakistan; 3 Biotechnology Department, Quaid-i-Azam University, Islamabad 44000, Pakistan; 4 Pediatric Cardiology Department, Hayatabad Medical Complex, Peshawar 25000, Pakistan; 5 Princess Al-Jawhara Albrahim Center of Excellence in Research of Hereditary Disorders, King Abdulaziz University, Jeddah 80205, Kingdom of Saudi Arabia

**Keywords:** Congenital generalized lipodystrophy, *BSCL2*, Deletion mutation, Pakistani population

## Abstract

**Background:**

Congenital generalized lipodystrophy (CGL) also known as Berardinelli-Seip Congenital Lipodystrophy (BSCL) is a genetically heterogeneous disorder characterized by loss of adipose tissues, Acanthosis nigricans, diabetes mellitus, muscular hypertrophy, hepatomegaly and hypertriglyceridemia. There are four subclinical phenotypes of CGL (CGL1-4) and mutations in four genes *AGPAT2, BSCL2, CAV1* and *PTRF* have been assigned to each type.

**Methods:**

The study included clinical and molecular investigations of CGL disease in a consanguineous Pakistani family. For mutation screening all the coding exons including splice junctions of *AGPAT2, BSCL2, CAV1* and *PTRF* genes were PCR amplified and sequenced directly using an automated DNA sequencer ABI3730.

**Results:**

Sequence analysis revealed a single base pair deletion mutation (c.636delC; p.Tyr213ThrfsX20) in exon 5 of *BSCL2* gene causing a frame shift and premature termination codon.

**Conclusion:**

Mutation identified here in *BSCL2* gene causing congenital generalized lipodystrophy is the first report in Pakistani population. The patients exhibited characteristic features of generalized lipodystrophy, Acanthosis nigricans, diabetes mellitus and hypertrophic cardiomyopathy.

**Virtual Slides:**

The virtual slide(s) for this article can be found here: http://www.diagnosticpathology.diagnomx.eu/vs/1913913076864247.

## Background

Congenital generalized lipodystrophy (CGL), or Berardinelli-Seip congenital lipodystrophy (BSCL), is a genetically heterogeneous disorder characterized by loss of adipose tissues and sub-cutaneous fats, enlarged fatty liver, hypertrophic muscles, Acanthosis nigricans, increased serum triglyceride level, insulin intolerance or diabetes mellitus. Pathogenicity of four genes has been reported in four clinically overlapping phenotypes so far.

The affected individuals of congenital generalized lipodystrophy type 1 (CGL1, MIM 608594) are characterized by typical poor fat accumulation in the metabolically active and mechanical adipose tissues. In addition these patients have special features of lytic bone lesions that are absent in other forms of CGL [1,2]. Loss-of-function mutations in 1-acylglycerol-3-phosphate-O-Acyltransferase (*AGPAT2*, MIM 603100) gene at chromosome 9q34.3 have been assigned to cause CGL1 phenotype [3,4].

Pathogenic variants of homologous to mouse gamma-3-linked (*BSCL2*, MIM 606158) gene, located at chromosome 11q13, cause congenital generalized lipodystrophy type 2 (CGL2, MIM 269700) or a seipin-deficient phenotype [3,5]. The subcutaneous biopsies examinations of patients have revealed scattered groups of small adipocytes with low but detectable lipid content [6]. The seipin-deficient individuals have generalized congenital lipodystrophy, hypertrophic cardiomyopathy, higher rates of mild mental retardation and an earlier onset of diabetes [7-9].

Mutations in caveolin 1 (*CAV1*, MIM 601047) gene, located at chromosome 7q31.3, cause congenital generalized lipodystrophy type 3 (CGL3, MIM 612526). Caveolin-1-deficient patients exhibit unique manifestation of short stature, hypocalcemia and vitamin D resistance [10]. Recently another form congenital generalized lipodystrophy type 4 (CGL4, MIM 613327) has also been described caused by mutations in RNA polymerase 1 and transcript release factor (*PTRF*, MIM 603198) gene at chromosome 17q21 [11].

In the present study clinical and molecular analysis of a four generations consanguineous Pakistani family demonstrating autosomal recessive CGL phenotype was performed. Direct sequencing of four candidate genes in a Pakistani family revealed a single base pair deletion mutation in *BSCL2* gene.

## Methods

### Human subjects

**Figure 1:**
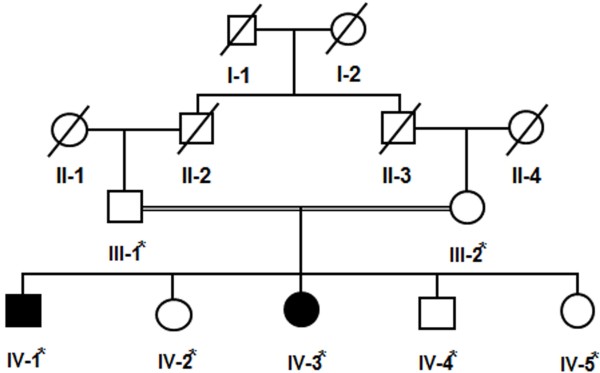
**Pedigree drawing of the family exhibiting congenital generalized lipodystrophy with autosomal recessive mode of inheritance.** Cousin marriage is denoted by double line between the couple. Samples available for DNA analysis are marked with asterisk.

The present four-generation family, exhibiting features of CGL or BSCL (Figure 1), belonged to Peshawar city of Khyber Pakhtunkhwa province, Pakistan. The affected individuals were born to first degree cousins, suggesting that affected individuals are homozygous for a mutant allele. All affected and unaffected individuals underwent examination at the local government hospitals. Prior to start of study, approval was obtained from Institutional Review Board (IRB), Khyber Medical University Peshawar, Pakistan. Informed consent for the study including presentation of photographs was obtained from affected individuals and their parents for publication. Genomic DNA extraction and polymerase chain reaction was performed as described earlier [12].

### Mutational analysis

Entire coding region and splice junction sites of *AGPAT2, BSCL2, CAV1* and *PTRF* genes were amplified by PCR and screened by DNA sequencing for potential sequence variants. Primer sequences (available on request) were designed for each exon using Primer3 software [13] and checked for specificity using Basic Local Alignment Search Tool (BLAST; http://www.ncbi.nlm.nih.gov/blast). Purification of the PCR-amplified DNA was performed with commercially available kits (Marligen Biosciences, Rockville MD, USA). Sequencing of the potential candidate genes was performed using Big Dye Terminator v3.1 Cycle Sequencing Kit, together with an ABI Prism 3730 Genetic Analyzer (Applera, Foster City, CA, USA). Sequence variants were identified via Bioedit sequence alignment editor version 6.0.7 (http://www.mbio.ncsu.edu/BioEdit/bioedit.html).

## Results

### Clinical features of the affected individuals

#### Skin

**Figure 2:**
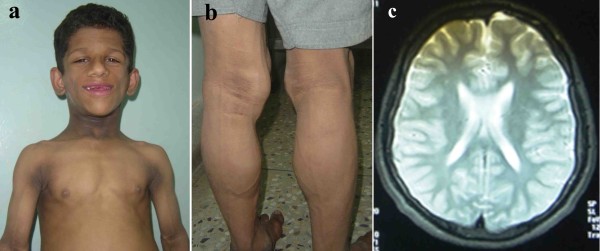
**Clinical features of the patient (IV-1) with congenital generalized lipodystrophy.** (**a**) Note curly scalp hair, hypodontia, abdominal distension, acanthosis nigricans, velvety thickening and hyperpigmentation of the skin around neck, in axillae and antecubital fossae (**b**) Hypertrophic muscles over calves and acanthosis nigricans in the popliteal fossae (**c**) Magnetic Resonance Imaging showing no exaggeration of the external or internal cerebrospinal fluid (CSF) spaces suggesting that brain atrophy is not evident.

The affected members (IV-1 and IV-3) of the family showed characteristic features of congenital generalized lipodystrophy. Both the affected individuals had acanthosis nigricans (velvety thickening and hyperpigmentation of the skin) which was more prominent around neck and in body folds including axillae, anticubital fossae and popliteal fossae. Both the affected individuals had prominent veins, rough dry skin and umbilical protrusion. Curly and dry hair was present with mild hypertrichosis over the scalp (Figure 2a).

#### Musculoskeletal examinations

Muscular hypertrophy was observed in skeletal muscles more prominently at arms and shin areas in both the affected individuals (Figure 2b). Radiological examinations of bones in the affected individuals showed relatively hyper-density of metacarpals, phalanges, sclerotic hip bones and adjacent portion of both femurs. Slight hyper-density was observed in skull bone, however, vault thickness and para-nasal sinuses were normal in both the affected individuals. Chest X-rays revealed normal lung fields, normal cardiac shadow with slight hyper-density of scapulae, humerus and clavicles. Magnetic Resonance Imaging examination (MRI) of the brain does not reveal generalized brain atrophy (Figure 2c).

#### Abdominal and cardiac examinations

Ultrasonography of the abdomen revealed moderate hepatomegaly with mild splenomegaly in both the affected individuals. There was no symptom of polycystic ovarian syndrome in 7 years affected female (VI-3), however her 11 years affected brother (VI-1) had moderate genital enlargement. Both the patients exhibited hypertrophic cardiomyopathy. Electrocardiography (ECG) of both the affected individuals showed sinus rhythm and right bundle branch pattern. Echocardiography of IV-1 revealed patent foramen ovale and mild left ventricular hypertrophy with intact inter-ventricular septum, while IV-3 showed small secundum atrial septal defect with left to right shunt, minimal right ventricular dilatation, and an intact inter-ventricular septum. There was no significant atrio-ventricular valve re-gurge. The left ventricular function was found normal (data not shown).

#### Biochemistry tests

The total serum bilirubin and serum electrolytes levels of both the affected individuals were within the normal range. Serum glutamate pyruvate, blood sugar, alkaline phosphatase and triglyceride levels were raised in both the affected individuals. High density lipoprotein levels were low in both the affected individuals than normal in both of them. The glycosylated hemoglobin (HbA_1_C) level of the affected individual IV-1 was slight higher than controlled diabetic range while that of IV-3 was in the non-diabetic level (Table 1).

**Table 1:** Biochemistry profile of the affected individuals

**S. No.**	**Test**	**Patient VI-1**	**Patient VI-3**
	**Liver function**		
1	Serum bilirubin	0.7 mg/dl	0.9 mg/dl
2	Serum glutamate pyruvate transferase	143 U/L	135 U/L
3	Alkaline phosphatase	911 U/L	717 U/L
	**Lipid profile**		
4	Triglyceride	394 mg/dl	406 mg/dl
5	Cholesterol	155 mg/dl	196 mg/dl
6	High density lipids	14 mg/dl	34 mg/dl
7	Low density lipids	77 mg/dl	129 mg/dl
	**Serum electrolytes**		
8	Na^+^	141	144
9	K^+^	3.71	3.99
10	Cl^-^	105.1	108.5
	**Sugar level**		
11	Fasting serum glucose	124 mg/dl	51 mg/dl
12	HbA_1_C	6.5%	5.6%

### Study limitations

The patients did not cooperate for tissue biopsy therefore histo-pathological examinations of the skin and sural nerves were not performed.

### Mutation screening

**Figure 3:**
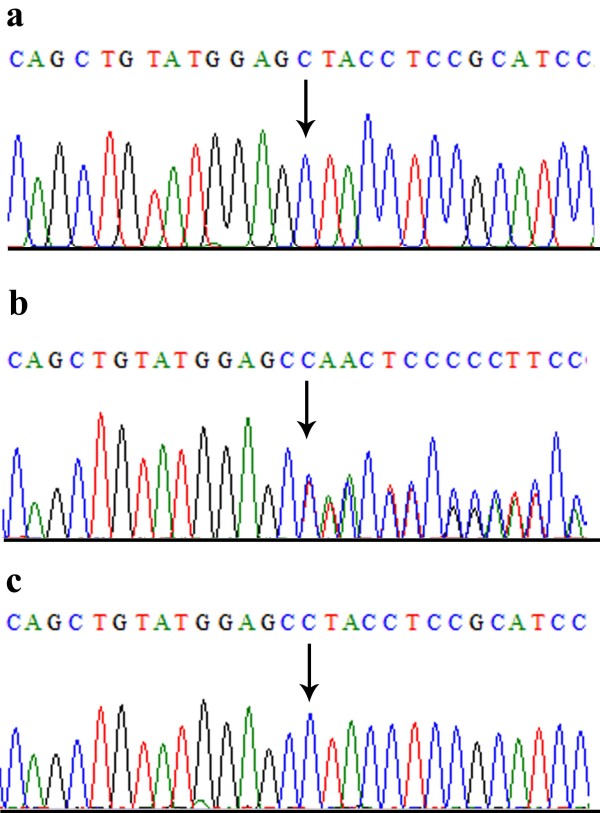
**Sequence analysis of a single base pair deletion mutation in *BSCL2 *gene (c.636delC).** (**a**) DNA sequencing of exon 5 in an affected individual IV-1 (**b**) parent or carrier III-1 (**c**) unaffected or normal individual.

To search for potential sequence variants, all coding exons and splice junction sites of *AGPAT2, BSCL2, CAV1* and *PTRF* genes were sequenced initially in two affected and one unaffected individuals of the family. A homozygous deletion mutation of a single base cytosine at complementary DNA position 636 (c.636delC) was detected in exon 5 of *BSCL2* gene in both the affected individuals (Figure 3). This deletion probably shifted the reading frame leading to a premature stop codon and adding 20 non-specific amino acid residues BSCL2 protein (p.Tyr213ThrfsX20). Mutation analysis of *BSCL2* exon 5 revealed c.636del in heterozygous state in obligate carriers and phenotypically unaffected individuals of the family. To ensure that c.636delC does not represent a neutral polymorphism in this population, a panel of 100 unrelated and ethnically matched control individuals was screened for this mutation, thus confirming that mutation was not present outside the family.

## Discussion

Loss-of-function mutations in the *BSCL2* gene cause a severe form of lipodystrophy, whilst characteristic gain-of-function mutations are believed to be associated with aggregation of unfolded protein in endoplasmic reticulum resulting in neurodegeneration leading to a heterogeneous group of neuropathies [5]. The study presented here was a four generations consanguineous family, originated from Peshawar city Pakistan. Clinical features of the affected individuals resembled CGL2 phenotype. Moreover the first degree consanguinity of the parents and autosomal recessive mode of inheritance of the disease phenotype was a definite clue for a homozygous mutation. Mutations in *BSCL2* leading to CGL2 phenotype have been identified worldwide [5,14-16].

The generalized loss of adipose tissue, increased triglyceride levels and steatosis of the liver were comparable to a homozygous mutation p.Tyr213ThrfsX20 identified in an Indian family [5]. Early onset diabetes mellitus present in our patients were comparable with homozygous nonsense mutations identified in Chinese and severe insulin resistance in Japanese and Brazilian patients [9,14,15], however we did not perform the insulin resistance test in our patients. The hypertrophic cardiomyopathy identified in our cases was less severe as compared to Chinese patient caused by homozygous nonsense mutation [17]. There was mild mental retardation (IQ score 65–75) in both of our patients but the brain MRI did not reveal brain atrophy.

The variable features of neuropathies are mostly associated with heterozygous mutations in *BSCL2* dHMN, Charcot-Marie-Tooth (CMT) and Silver syndromes [16,18,19]. In 2005, based on disease allele penetrance and severity of phenotype caused by heterozygous mutations in *BSCL2* gene p.N88S in Austrian and German families, these patients were classified into six subclinical groups with overlapping features of HMN, CMT and Silver syndrome [20]. However, in 2009, Brusse *et al*. discovered a digenic inheritance of hereditary motor neuropathy in a large Dutch family with heterozygous mutation p.N88S in the *BSCL2* gene and segregating autosomal dominant disease haplotype at chromosome 16p [21]. The phenotypic variability ranged from strictly neuropathic weakness to a spastic paraplegia with hereditary motor neuropathy presenting clinical phenotype similar to Silver syndrome [21]. Very recently another heterozygous mutation p.S90W identified in two Korean CMT type 2 disease patients [22] was associated with increased density of myelinated fibers. However, this feature was slightly different from the previously reported mutation p.S90L in three Italian patients representing CMT type 2 phenotype with pyramidal signs and subclinical sensory involvement on sural nerve biopsy [23].

Human *BSCL2* gene encodes 398 or 462 amino acids seipin protein from either of the three transcripts 1.6 kb, 1.8 kb and 2.2 kb [24]. The 1.8 kb transcript is exclusively expressed in brain and testis while the other two are ubiquitously expressed [18]. Seipin protein is located in endoplasmic reticulum acting as a regulator of lipid catabolism and is essential for the differentiation of fat cells [18,24]. Studies have revealed role of seipin in proper lipid storage and regulation of cAMP/PKA-mediated lipolysis in adipose differentiation [25,26]. In adult mice brain *bscl2* expression studies have suggested the possible involvement of seipin in central regulation of energy balance.

According to UniprotKB database (http://www.uniprot.org/uniprot/Q96G97), 398 amino acids seipin protein has five domains including 2 cytoplasmic [1-26aa and 264-398aa], 2 transmembrane [27-47aa and 243-263aa] and a luminal [48-242aa]. So far 31 mutations are reported in *BSCL2* gene including 12 missense/nonsense, 4 small insertions, 4 small deletions, 7 splice-site mutations and 4 complex rearrangements causing related phenotypes as summarized in Table 2.

**Table 2:** **List of mutations in *BSCL2 *gene so far**

**Exon**	**cDNA**	**Protein**	**Phenotype**	**References**
	**Missense/Nonsense**			
2	c.232A>G	p.Thr78Ala	CGL2	[27]
2	c.263A>G	p.Asn88Ser	dHMN	[16,18,28]
2	c.269C>T	p.Ser90Leu	dHMN, CMT2	[18,29,30]
2	c.269C>G	p.Ser90Trp	dHMN, CMT2	[22]
2	c.272T>C	p.Leu91Pro	CGL2	[27]
3	c.412C>T	p.Arg138X	CGL2	[5]
4	c.560A>G	p.Tyr187Cys	CGL2	[15]
4	c.565G>T	p.Glu189X	CGL2	[14]
5	c.634G>C	p.Ala212Pro	CGL2	[5,25]
6	c.684C>G	p. Tyr228X	CGL2	[7]
7	c.823C>T	p.Arg275X	CGL2	[31]
10	c.1171C>T	p.Gln391X	CGL2	[27]
	**Insertion**			
1	c.154_155insTT	p.Tyr53SerfsX39	CGL2	[7]
3	c.301_302insAA	p.Met101LysfsX10	CGL2	[5]
3	c.325insA	p.Thr109AsnfsX5	CGL2	[5]
6	c.782dupG	p.Ile262HisfsX12	CGL2	[7]
	**Deletion**			
3	c.315_316delGT	p.Tyr106SerfsX7	CGL2	[5]
3	c.317_321delATCGT	p. Tyr106CysfsX6	CGL2	[5,25]
5	c.636delC	p.Tyr213ThrfsX20	CGL2	[5]
5	c.652_662del11	p.Ala218TrpfsX51	CGL2	[32]
	**Splice-site**			
IVS2 -11A>G	Exon skipping	Protein truncation	CGL2	[33]
IVS4 +1G>A	Exon skipping	Protein truncation	CGL2	[5]
IVS5 -2A>G	Exon skipping	Protein truncation	CGL2	[27]
IVS5- 2A>C	Exon skipping	Protein truncation	CGL2	[34]
IVS6 +5G>A	Exon skipping	Protein truncation	CGL2	[5]
IVS6 -3C>G	Exon skipping	Protein truncation	CGL2	[5,25]
IVS6 -2A>G	Exon skipping	Protein truncation	CGL2	[7]
	**Complex rearrangements**			
1	c.192_193delCCinsGGA		CGL2	[5,25]
1	c.193delCinsGGA		CGL2	[7]
4-6	Deletion of exons 4-6		CGL2	[5]
5-6	Indel leading exons 5–6 deletion		CGL2	[5]

dHMN= distal motor hereditary neuropathy; CMT2 = Charcot-Marie-Tooth type 2; CGL2 = Congenital generalized lipodystrophy type 2.

More than three hundred cases of BSCL have been reported in the medical literature with an estimated prevalence of 1 in 10 million people in USA [35]. However this condition is more common in other populations around the world like Lebanon, Brazil, Portugal and Sultanate of Oman with an estimated prevalence of 1:25000 to 1:1000000 [36]. The incidence data of BSCL and many other rare disorders are not available from Pakistan partly due to lack of disease registry database systems and non-availability of neonatal screening programs in the hospitals.

Generally, in families segregating autosomal recessive disorders, high frequency of consanguineous marriages may increase the frequency of homozygotes in the population leading to increased incidence of certain lesions, their founder effect and also the appearance of new mutations [37]. Although most causative mutations in Mendelian diseases are reported in single families, certain mutations may occur more frequently in some populations than others. Few examples of such prevalent mutations may be observed in Wilson’s disease [38], hemophilia [39], Hurler disease [37] and thalassemia [40]. Knowledge of the differences in the worldwide distribution of particular mutations may help to design shortcuts for genetic diagnosis and screening of relevant inherited diseases.

## Conclusion

The identified mutation c.636delC (p.Tyr213ThrfsX20) in exon 5 of *BSCL2* gene is the first report of CGL2 from Pakistan. The current study extends the body of evidence that describes the role of *BSCL2* in congenital generalized lipodystrophy and association of loss-of-function mutations with severe lipodystrophy phenotype. The clinical features in our patients are similar to CGL2 phenotype but lack of motor neuropathies that are usually associated with Silver syndrome and Charcot-Marie-Tooth disease were not observed in our case. Understanding the molecular defects of *BSCL2* gene in the patients can be helpful in the genetic counseling and prenatal diagnosis of affected families and may help to improve specific therapeutic interventions.

## Competing interest

The authors declare that they have no competing interests.

## Authors’ contributions

OR performed the molecular studies, NK and KK performed clinical diagnosis, JA performed radiological examination and data interpretation, MAK performed sequencing alignment and manuscript writing, JYA and MN critically reviewed the manuscript, MJ planned the study and finalized the manuscript. All authors agreed upon the final manuscript.
